# Review of thyroid cytopathology: An atlas and text

**Published:** 2008-10-03

**Authors:** Melina Flanagan, Prabodh Gupta

**Affiliations:** Department of Pathology, West Virginia University, Morgantown, WV, USA; 1 Department of Pathology, Hospital of the University of Pennsylvania, 3400 Spruce St, 6.093 Founders Building, Philadelphia, PA 19104, USA

## Book Review

Dr. Kini's Thyroid Cytopathology: An Atlas and Text essentially serves as the third edition of Thyroid (Guides to Clinical Aspiration Biopsy). Arriving twelve years after the second edition, this book has a new format. It has been substantially updated with a decade's worth of new data, and contains hundreds of color photographs.

Thyroid Cytopathology has 23 chapters. There are discussions on the techniques of fine needle aspirations (FNA) and cytopreparation, adequacy, and guidelines for FNA assessment and categorization, in several chapters. Another 14 chapters are each dedicated to a particular entity within thyroid pathology (nodular goiter, follicular adenoma and carcinoma, Hürthle cell lesions, papillary carcinoma, insular carcinoma, anaplastic carcinoma, medullary carcinoma, thyroiditis, lymphoma, thyroid carcinomas metastatic to other sites, metastases to the thyroid, cystic lesions, spindle cell lesions, and miscellaneous lesions). Additional chapters focus on ancillary techniques (with particular focus on immunohistochemistry and molecular techniques) and the application of thyroid aspirations on management decisions.

The text and atlas provide a comprehensive guide to thyroid cytopathology. There are over 1,700 full-color photomicrographs of excellent quality. Some of these are the same photographs which were printed in half-tone in the second edition. For each disease entity, there are several photographs of both aspirations and surgical pathology, demonstrating a range of features. In fact, there is sufficient cytohistologic correlation in both images and text, giving out the message that although this book is called an atlas of thyroid cytopathology, it is more precisely an atlas of thyroid pathology with a focus on cytopathology! The discussion of each entity includes cytopathologic characteristics, differential diagnoses and diagnostic pitfalls, as well as clinical information regarding the likelihood of histologic outcomes for each cytologic diagnosis.

One shortcoming of the book is that liquid-based cytopathology, which is being used for thyroid aspirates in more and more institutions, is discussed only in one dedicated Appendix. The advantages and disadvantages of this technique are given their full due, and images of the more common disease entities are included. However, given that this preparation is gaining prevalence, I would have preferred to have the discussion of the technique included in the initial chapter on cytopreparatory techniques, and the images incorporated into the chapters on each entity.

Dr. Kini's approach to both follicular and Hürthle cell lesions differs from conventional wisdom in that she differentiates between adenomas and carcinomas on thyroid aspirations. The chapters on these entities contain extensive discussion regarding this distinction, as well as numerous images. While some readers may find this useful, it may make others leery.

In summary, Thyroid Cytopathology: An Atlas and Text is a highly valuable diagnostic reference. In addition, it is very readable, and almost conversational. Dr. Kini presents facts and statistics, as well as controversial food for thought (‘Should we really consider surgical pathology the gold standard?) in an educational and entertaining manner. We would recommend this book to pathologists of all levels, who are interested in learning about or increasing their knowledge bank in the field of thyroid pathology.

